# Implementing Voluntary Medical Male Circumcision for HIV Prevention in Nyanza Province, Kenya: Lessons Learned during the First Year

**DOI:** 10.1371/journal.pone.0018299

**Published:** 2011-04-04

**Authors:** Amy Herman-Roloff, Emma Llewellyn, Walter Obiero, Kawango Agot, Jeckoniah Ndinya-Achola, Nicholas Muraguri, Robert C. Bailey

**Affiliations:** 1 Division of Epidemiology and Biostatistics, University of Illinois at Chicago, Chicago, Illinois, United States of America; 2 Nyanza Reproductive Health Society, Kisumu, Kenya; 3 Impact Research and Development Organization, Kisumu, Kenya; 4 Department of Medical Microbiology, University of Nairobi, Nairobi, Kenya; 5 National AIDS and STI Control Programme, Nairobi, Kenya; Johns Hopkins University Bloomberg School of Public Health, United States of America

## Abstract

**Background:**

In 2007, the World Health Organization endorsed male circumcision as an effective HIV prevention strategy. In 2008, the Government of Kenya (GoK) launched the national voluntary medical male circumcision (VMMC) program in Nyanza Province, the geographic home to the Luo, the largest non-circumcising ethnic group in Kenya. Currently, several other African countries are in the early stages of implementing this intervention.

**Methods and Results:**

This paper uses data from a health facility needs assessment (n = 81 facilities) and a study to evaluate the implementation of VMMC services in 16 GoK facilities (n = 2,675 VMMC clients) to describe Kenya's experience in implementing the national program. The needs assessment revealed that no health facility was prepared to offer the minimum package of services as outlined by the national guidelines, and partner organizations were called upon to fill this gap. The findings concerning human resource shortages facilitated the GoK's decision to endorse trained nurses to provide VMMCs, enabling more facilities to offer the service. Findings from the evaluation study resulted in replacing voluntary counseling and testing (VCT) with provider-initiated testing and counseling (PITC) and subsequently doubling the proportion of VMMC clients tested for HIV.

**Conclusions:**

This paper outlines how certain challenges, like human resource shortages and low HIV test rates, were addressed through national policy changes, while other challenges, like large fluctuations in demand, were addressed locally. Currently, the program requires significant support from partner organizations, but a strategic plan is under development to continue to build capacity in GoK staff and facilities. Coordination between all parties was essential and was facilitated through the formation of national, provincial, and district VMMC task forces. The lessons learned from Kenya's VMMC implementation experience are likely generalizable to other African countries.

## Introduction

Male circumcision (MC) is the surgical removal of the penis foreskin and is practiced around the world for medical, religious, and cultural purposes. The 2007 Kenya AIDS Indicator Survey reported that 85% of Kenyan men were circumcised and 7.4% of the population was HIV positive [1]; the 2009 Kenya Demographic and Health Survey reported that 86% of Kenyan men were circumcised and 6.3% of the population was HIV positive.[2] However, heterogeneity in HIV and MC prevalence by geography and ethnic group is evident; in Nyanza Province, the geographic home to the traditionally non-circumcising Luo ethnic group, 21.5% of Luo men are circumcised and 17.1% tested positive for HIV.[2]

To date, over 40 observational studies, and three randomized controlled trials (RCTs), have established that MC reduces the risk of HIV acquisition in heterosexual men by approximately 60%.[3]–[6] These positive findings prompted the World Health Organization (WHO) and the Joint United National Programme of HIV/AIDS (UNAIDS) to release a policy statement in 2007 recommending that medical MC be implemented as one component of a comprehensive HIV prevention strategy in regions with low MC rates, high HIV prevalence, and where heterosexual sex is the primary mode of transmission.[7]

In response to consistent research findings and the WHO/UNAIDS policy statement, the Government of Kenya (GoK) launched a national Voluntary Medical Male Circumcision (VMMC) program in November, 2008. The GoK plans to circumcise 860,000 men during the next five years, and partner organizations were invited to support the GoK by implementing program activities and building capacity during the initial phase.[8] Since the launch of the program, over 230,000 MCs have been performed by GoK staff and partners.

This paper uses data from a facility needs assessment and a study evaluating the roll-out of the VMMC program to elucidate challenges, responses, and lessons learned during the first year of implementing the national VMMC program in Nyanza Province, Kenya. While this is not intended to be a full report, it is the first evaluation, including recommendations and possible solutions, to be published. Our experience is likely to be useful to several African countries, with health delivery systems similar to Kenya, which are in the early stages of implementing comparable programs.

## Methods

### Ethics Statement

The health facility needs assessment was conducted through the National AIDS and STI Control Programme to support the Kenya Ministry of Health and to inform policy and resource allocation at the launch of the VMMC program. Since the facility assessment was conducted for administrative purposes, and not research, no approval was sought from ethical review committees in- or out-side of Kenya. All evaluation study research staff completed the online Collaborative Institutional Training Initiative training course on human subject protection. Written consent (adults) or assent and parental permission (minors) was obtained from all study participants. The evaluation study was approved by the University of Illinois at Chicago Institutional Review Board in Chicago, USA (protocol: 2007-0913), and the Kenyatta National Hospital Ethics and Research Committee, Nairobi, Kenya (protocol: P338/11/2007).

### Health Facility Needs Assessment

The data presented in this paper come from two sources: 1) a health facility needs assessment and 2) a study evaluating VMMC implementation in GoK health facilities. All data were collected in Kisumu and Nyando districts in Nyanza Province, Kenya.

The health facility needs assessment was conducted February-October, 2008, prior to the launch of the national VMMC program. All health facilities in the two districts were surveyed (n = 81) using a 3-page questionnaire. The in-charge clinician, or designee, was interviewed and the resources available for VMMC were visually observed by the assessment team. The assessment focused on the seven minimum criteria for MC service provision as outlined in the Kenya national clinical manual including:[9]

1. Room available for surgery (e.g., minor theater)
2. Room available for recovery
3. Trained and available staff
4. Sterilization and infection control compliance
5. HIV voluntary counseling and testing (VCT) and risk-reduction counseling
6. STI syndromic diagnosis and treatment
7. Provision and promotion of male and female condoms

### Evaluation Study

The evaluation study was conducted in 16 GoK health facilities to assess the implementation of the program through monitoring VMMC clients from pre-procedure through 40-days post-MC. VMMC clients, aged ≥12 years, who voluntarily elected to be circumcised and gave their written informed consent/assent to participate in the study, were eligible to enroll in the study (n = 2,675). Data were analyzed using SAS version 9.1; descriptive results are presented here.

## Results

**Figure 1:**
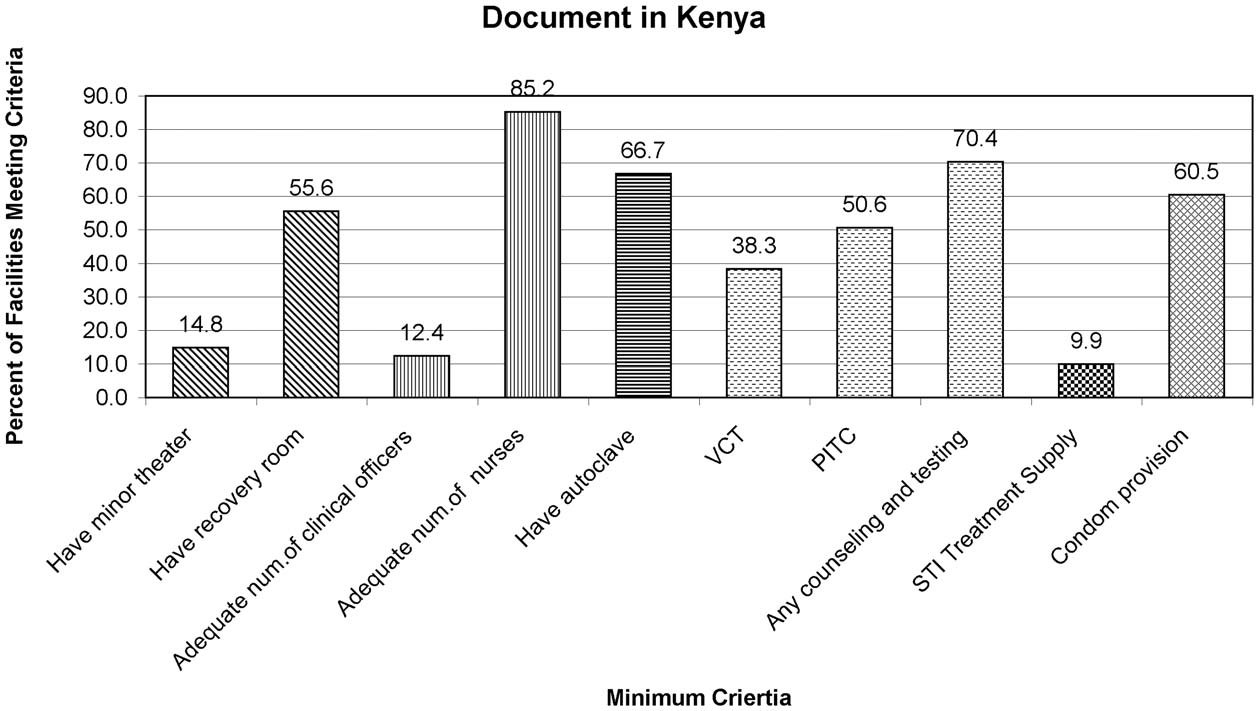
Percent of Government of Kenya Health Facilities in Kisumu and Nyando Districts with Components of the Minimum Criteria for VMMC Service Provision as Outlined in the National Guidance Document in Kenya.

Eighty-one health facilities were assessed, and no facility possessed all seven VMMC criteria necessary to provide safe services (Figure 1).

### Infrastructure

#### Challenge

Most facilities had inadequate infrastructure. Few (14.8%) had an existing minor theater; 64.2% had space for a minor theater to be renovated; and 21% had no space for a minor theater. Similarly, 35% of facilities had no space available for a recovery room even if renovations were provided.

#### Response and Lessons Learned

Renovations were provided to six GoK facilities by partner organizations. These facilities were chosen after conducting a spatial analysis considering proximity to major roads, availability of staff to provide MC services, and size of population in catchment area; a well-accessed facility, with adequate client flow was also considered. Renovations included partitioning, painting, minor repairs, installing doors and windows, plumbing, and electrical work. The cost of renovations varied by the amount of work that needed to be done at each facility (range: $650–$7,000).

### Equipment and Supplies

#### Challenge

Most facilities were not prepared to provide VMMC services without the provision of equipment and supplies from partner organizations. For example, nearly all facilities reported providing syndromic STI management (96.3%), but based on visual observation, only 9.9% had an adequate STI drug supply. Additionally, while GoK facilities committed to providing HIV test kits for the VMMC program, “stock outs” occurred frequently which negatively impacted HIV testing uptake.

#### Response and Lessons Learned

Necessary equipment and supplies were provided to GoK facilities including: autoclaves, surgical couches, lamps, trays, trolleys, linens, privacy curtains, reusable surgical packs, and consumables. The cost of consumables per procedure was $14.67. Since the GoK committed to providing HIV test kits, partner organizations sought authorization to request kits directly from the national supplier in order to closely monitor and manage the stock in the field. The national medical supply agency plays an integral role in the implementation of a surgical HIV prevention intervention, and should be engaged early in the scale-up process, even if consumables are being supplied in the short-term, since it can take considerable time to coordinate the distribution of supplies and to add VMMC supplies to the Essential Drugs and Supplies List.

### Human Resources

#### Challenge

At the time of the assessment, only medical officers and clinical officers were authorized to provide VMMC services in Kenya. There were few medical officers in the study districts (n = 8), and 12.4% of facilities had an adequate numbers of clinical officers to offer VMMC services (≥2 clinical officers). The assessment revealed that 85.2% of facilities had an adequate number of nurses to provide VMMC services, but at the time of the assessment, nurses were not legally approved to provide VMMC services.

#### Response and Lessons Learned

Conducting an assessment informed our understanding of the health staffing shortage, which plagues many African countries, and supported the efforts of the national MC task force to lobby for trained nurses to be authorized to perform VMMC procedures. The GoK announced this authorization on June 17, 2009. Despite the resulting increase in the VMMC workforce, GoK staff continue to struggle to incorporate VMMC into their routine duties mostly because staff report that they are too busy to devote time to providing a non-emergency procedure. Although partner organizations have trained over 1,000 GoK staff (including nurses), few are able to provide VMMC services routinely. Among VMMC clients in the evaluation study, 12% were circumcised by GoK staff. There are several possible explanations for this level of MC service provision among GoK staff including: MC is not a GoK performance indicator and is not included on the routine health monitoring form, and this can result in a lack of motivation to provide MC services; partner organizations provided MC services for almost one year before the GoK officially launched the VMMC program resulting in the perception that program was partner-initiated and partner-led; and health facilities have an inadequate number of staff to provide routinely what is perceived as a non-emergency, preventive service. In accordance with the national strategic plan, the GoK continues to request that partner organizations provide services during this initial phase while increasing their efforts to build the capacity of GoK staff to sustain the national program.

### HIV Testing and Counseling

#### Challenge

At the time of the assessment, approximately 1/3 of facilities offered voluntary counseling and testing (VCT) services. When considering VCT and provider initiated testing and counseling (PITC) together, 70.4% of facilities were prepared to offer HIV counseling and testing along with VMMC services. During the first months of providing VMMC services, VCT was implemented, and acceptance was <25%. Finally, HIV testing and counseling, along with MC counseling, proved to be a time-consuming process, often lasting over one hour per client.

#### Response and Lessons Learned

Partner organizations worked with the GoK to provide the necessary training to replace VCT with a PITC approach, and testing rates have increased to over 60%. Additionally, an “HIV testing target” of 80% has been set for each VMMC-dedicated team, and performance evaluations include an assessment of this target. In order to improve counseling efficiency, without sacrificing quality, “group education” sessions were implemented to explain the risks and benefits of MC to clients of the same age group at one time. Group counseling is followed by individual counseling during which clients, in a private setting, can ask questions they have, be tested for HIV, and receive personalized risk reduction counseling. This adaptation to the counseling protocol has reduced the total counseling time per client by approximately 50%. While we considered offering HIV testing and counseling to only sexually active clients, the national PITC guidelines state that all clients receiving care at a health facility should be screened for HIV. Currently, there are no national guidelines for the provision of MC services to HIV-positive men. In order to avoid unnecessary stigma, males deemed clinically fit to undergo surgery, who do not show signs of the Acquired Immunodeficiency Syndrome (AIDS), are eligible to receive MC services.

### Service Delivery

#### Challenge

Kenya adapted WHO's “Manual for Male Circumcision Under Local Anesthesia” to fit the Kenya context, and VMMC service providers were trained using this manual.[9] While there was consensus that VMMC services should be provided by trained, medical staff, there was no evidence about how and where services should be provided (i.e., health care facilities versus mobile services in the community) or about how many VMMCs could be provided each day in high-demand settings. These gaps complicated target-setting, budgeting, and planning activities.

#### Response and Lessons Learned

Since partner organizations provided the majority of services during the first year, a variety of service provision strategies, including fixed site and mobile service provision, were implemented and evaluated. Over 50% of services were provided by mobile partner teams working in small, low-resourced health facilities that lacked resources to provide services on their own. Mobile services were also provided in high-demand settings (e.g., at secondary schools and in fishing villages). In these settings, one service provision team, consisting of one clinical officer, nurse, counselor, and hygiene officer, working on two surgical couches simultaneously, task-sharing (e.g., one provider finishes dressing the wound while the other moves to give anesthetic to the next client), and task-shifting (e.g., the hygiene officer completes the clinical record form as the provider dictates), with optimal client flow, can perform up to 20 procedures per day. In order to increase efficiency for mobile teams, drivers were trained to assist with cleaning and preparing surgical packs. While mobile services can have additional costs due to the transport required and the carriage of additional items such as tents, tables and chairs, they can be very effective in high demand settings when multiple teams can work together using high efficiency models.

### Data Monitoring System

#### Challenge

The VMMC program was launched in November, 2008, but national reporting tools were not launched until January, 2010. While members of the national and provincial MC task forces collaborated to develop the forms, this was a time-consuming process, and in the interim, partner organizations created their own, non-standardized forms, leading to difficulties in data aggregation.

#### Response and Lessons Learned

We recommend that providers agree on core indicators and data collection tools before launching the program (WHO and PEPFAR MC indicators provide a good starting place), to ensure the collection of comparable data on indicators of interest.

### Service Access

#### Challenge

Matching client demand with service provision has been difficult. For example, while the GoK strategy targets males aged ≥15 years of age, 29% of clients are younger than 15 years. The traditional age for MC in neighboring communities ranges from 8–15 years, so parents and adolescents of this age discuss MC and are habituated to circumcision at these ages. Additionally, some VMMC implementing partners are organizations targeting primarily youth, so mobilizing youth was a natural result of their other program activities. Also, demand varies with school holidays, fishing seasons, and planting or harvest seasons.

#### Response and Lessons Learned

The large turn-out of adolescents resulted in revising our training and techniques for counseling adolescents about sex and reproductive health as well as standardizing the process for providing preventive medical services to a minor which now requires written parental permission and minor client assent. While minors continue to seek and receive MC services, because circumcising young boys delays the impact of this intervention, and most new HIV infections occur in adult men, we are developing new strategies to increase the number of males aged ≥18 years seeking VMMC services, like partnering with large employers in the region, using a voucher system, and offering incentives to mobilizers who recruit men aged over 18 years.

Service provision plans must be highly flexible to respond to changes in demand. Several strategies have been implemented to respond to fluctuations in demand including: 1) the creation of a master list of trained staff who can be hired on a temporary basis during high-demand periods; 2) the formation of mobile, rapid response service teams that are not assigned to a site, but move freely to sites with high demand; and 3) the implementation of client prioritization schemes serving males aged ≥18 years first, followed by males aged 15–17 years, and then younger minors. Additionally, MC-dedicated staff are encouraged to take leave during periods of low-demand.

While there are efforts underway to improve the efficiency of the MC procedure itself, we have learned that efficiency depends more on optimal client flow and efficient counseling and screening than on the time required for the procedure itself. Ultimately, if the surgery is efficient, but insufficient clients are present the impact of the program is compromised. Similarly, if there are ample clients present, but the pre-surgical processes are inefficient and time-consuming, the program is compromised. To this end, efforts to increase efficiency should be aimed at increasing demand and improving counseling efficiency rather than focusing exclusively on improving the efficiency of the surgical component of the MC process.

## Discussion

This evaluation has summarized the challenges, responses, and lessons learned during the implementation of an effective VMMC program in Kenya which has circumcised over 230,000 males in less than two years.

Recommendations from the national VMMC program in Kenya that may benefit other African countries implementing comparable programs include the following:

- Renovations to facilities may be necessary to scale-up program activities, and these improvements can be expensive, so it is important to develop a system to identify and prioritize high-volume, high-impact facilities.
- Health facility needs assessments are recommended to inform data-based decision-making about necessary programmatic and policy changes (e.g., endorsing nurses to provide VMMC services after appropriate training).
- As a surgical intervention, VMMC programs require well-coordinated partnerships between multiple government agencies (medical licensing, medical supply, etc.) and partner organizations. In Kenya, this was facilitated by the formation of dynamic national, provincial, and district MC task forces that met monthly and were well-attended by GoK representatives and all partner organizations.
- VMMC programs must be responsive to the variation in demand. Strategies like creating a pool of trained staff that can be temporarily hired, dedicating mobile teams to move around to facilities with high demand, implementing client priority schemes, and encouraging MC-dedicated staff to take leave during low-demand periods have been effective in responding to fluctuations in demand.
- While the national VMMC program was well-supported by committed governmental and non-governmental entities at all levels, for the program to be sustainable, GoK staff working at health facilities must also be committed to the program and empowered to provide the service. To this end, additional GoK staff need to be trained, the provision of VMMC services needs to be added to their job descriptions, and barriers to service provision need to be explored.
- In order to evaluate the implementation of a national VMMC program, a data monitoring system should be implemented before services are provided.

Despite our findings and recommendations, gaps still remain in our understanding of how to move the program forward. To guide us, we are currently assessing the barriers that trained GoK providers experience in providing services. We are evaluating techniques to strengthen the referral system between VMMC service sites and all HIV testing sites, and we are exploring new strategies to ensure consistent client flow.
